# Supplementary figures and images for: Deep learning allows genome-scale prediction of Michaelis constants from structural features
Source: PLoS Biol. 2021 Oct 19;19(10):e3001402. doi: 10.1371/journal.pbio.3001402 (PMC8525774; doi:10.1371/journal.pbio.3001402)

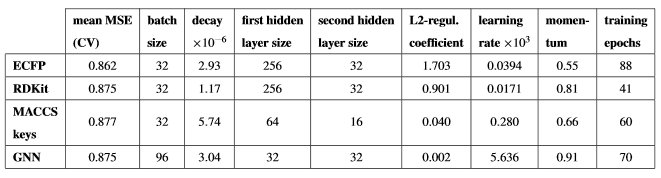

Supplement: S1 Table — The hypeparameter optimizations were performed for each of 4 different fingerprints of the substrates with a 5-fold cross-validation on the training set. (TIF) [file pbio.3001402.s001.tif]

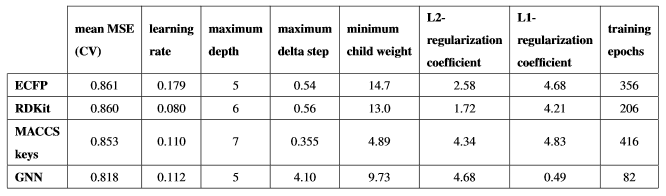

Supplement: S2 Table — The hypeparameter optimizations were performed for each of 4 different fingerprints of the substrates with a 5-fold cross-validation on the training set. (TIF) [file pbio.3001402.s002.tif]

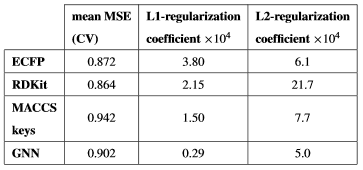

Supplement: S3 Table — The hyperparameter optimizations were performed for each of 4 different fingerprints of the substrates with a 5-fold cross-validation on the training set. (TIF) [file pbio.3001402.s003.tif]

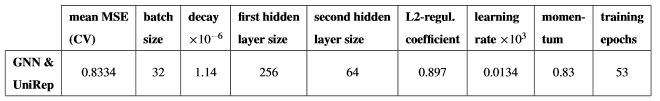

Supplement: S4 Table — The hypeparameter optimization was performed with a 5-fold cross-validation on the training set. (TIF) [file pbio.3001402.s004.tif]

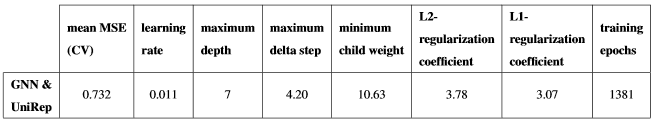

Supplement: S5 Table — The hypeparameter optimization was performed with a 5-fold cross-validation on the training set. (TIF) [file pbio.3001402.s005.tif]

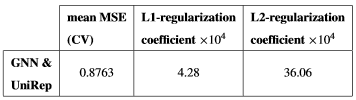

Supplement: S6 Table — The hyperparameter optimization was performed with a 5-fold cross-validation on the training set. (TIF) [file pbio.3001402.s006.tif]

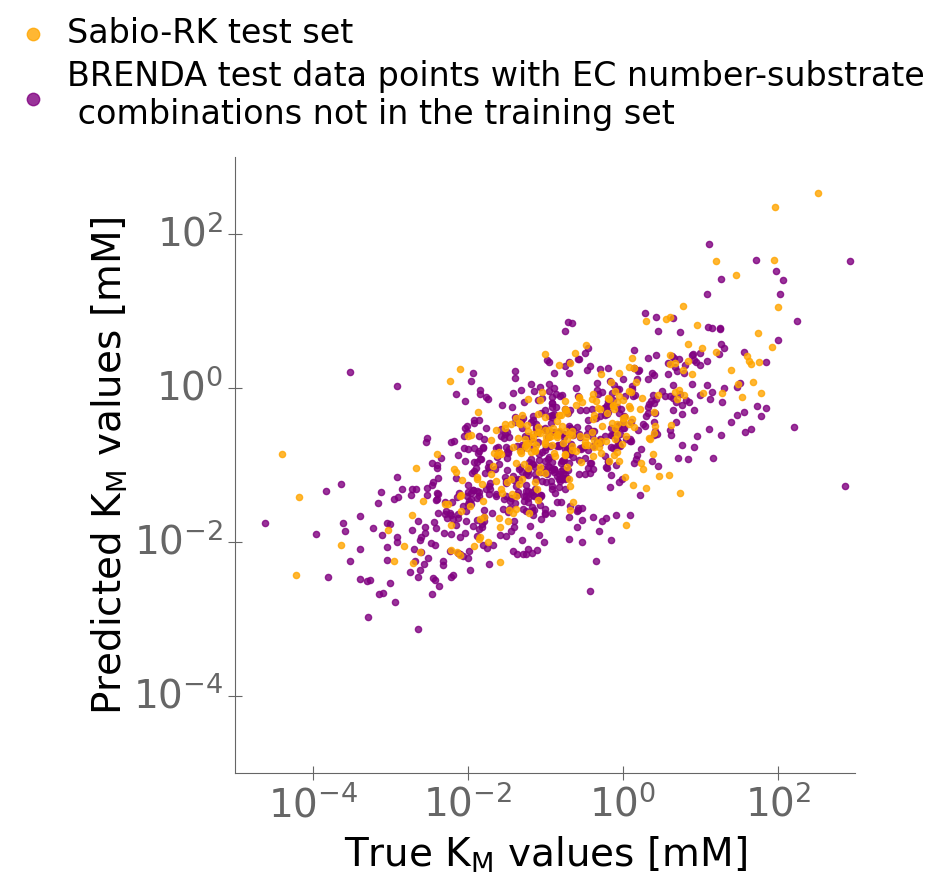

Supplement: S1 Fig — The scatter plot displays all data points of the Sabio-RK test set (orange) and all data points from the BRENDA test set with an EC number–substrate combination not present in the training set (violet). The data underlying the graphs shown in this figure can be found at https://github.com/AlexanderKroll/KM_prediction/tree/master/figures_data. (TIF) [file pbio.3001402.s007.tif]

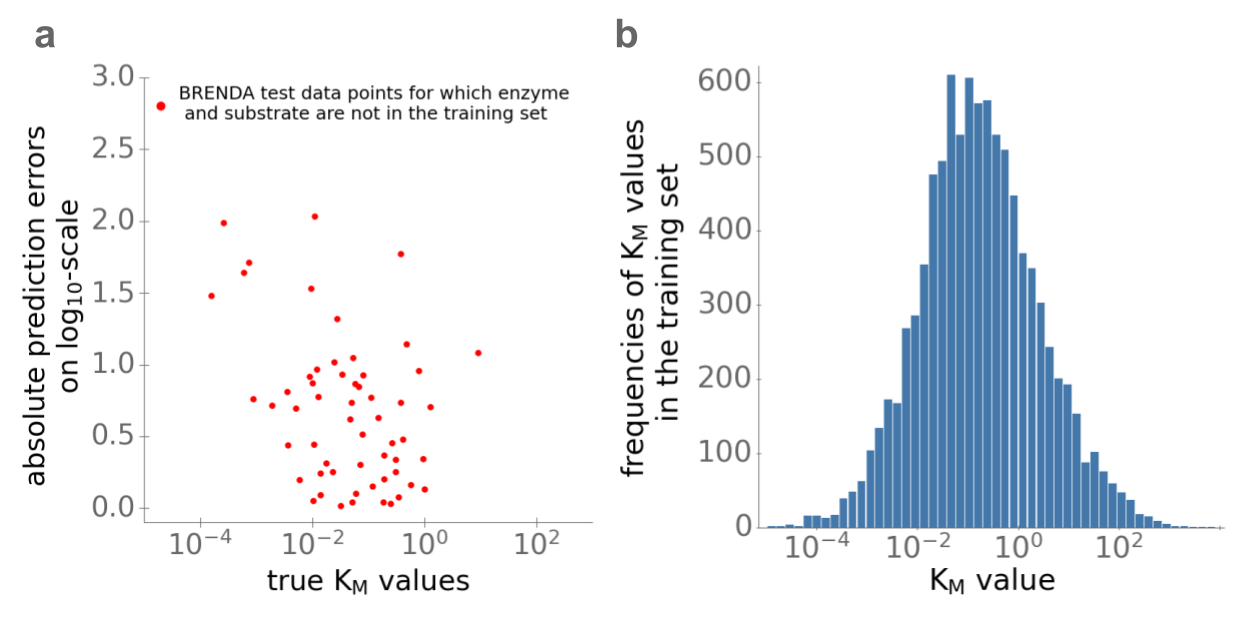

Supplement: S2 Fig — (a) Scatter plot of measured KM values and the absolute prediction errors of the BRENDA test data points for which neither the substrate nor the enzyme occurs in the training set. (b) Histogram with the distribution of the KM values in the training set. The data underlying the graphs shown in this figure can be found at https://github.com/AlexanderKroll/KM_prediction/tree/master/figures_data. (TIF) [file pbio.3001402.s008.tif]
