# Supplementary material for: Deep learning allows genome-scale prediction of Michaelis constants from structural features
Source: PLoS Biol. 2021 Oct 19;19(10):e3001402. doi: 10.1371/journal.pbio.3001402 (PMC8525774; doi:10.1371/journal.pbio.3001402)
Supplement: Attachment — Submitted filename: Kroll_Response2Reviewers.pdf [file pbio.3001402.s011.pdf]

Reviewer #1

1. *This paper describes several machine learning approaches to predict Km values. The most successful of these has an R^2 value of 0.42 between predicted and measured values for a test dataset. The predictions do not take into account experimental conditions (pH, temperature, ionic strength). They also assume that each enzyme has just one Km but allosteric enzymes, which are very common, have multiple Km values (1 per site) although a midpoint concentration corresponding to 0.5 Vmax can still be defined. Consequently, I'm skeptical whether the method is of value for researchers studying a specific system (e.g. as in drug development) but it could be helpful for large-scale modelling of metabolic fluxes, for example, when the details might matter less.*

Response: Our methodological revisions – in particular the use of deep protein representations (UniRep) for the enzymes – have led to a substantially improved accuracy, with R^2=0.53 on a test set; simulations indicate that this accuracy can be improved further once more experimental data becomes available. We agree with the Reviewer that for researchers studying a specific system, such as in drug development, more focused models will be more useful than our general approach. However, our genome-scale, organism-independent models are able to predict K_M values in cases where neither the enzyme family nor the substrate are represented in the training data, something that more specialized models could not do. We envisage that our predictions will be useful in particular for kinetic models of metabolism, and to provide a first rough estimate of an enzyme's overall K_M before experiments can be done.

Action: In the revised manuscript, we have clarified the above points, emphasizing the target applications of our model (last sentence of the abstract; l. 332; l. 353).

Reviewer #2:

1. *In the manuscript "Prediction of Michaelis constants from structural features using deep learning" by Kroll et. al. a graph-based method of predicting Km for a ligand is illustrated utilizing both chemical descriptors as well as a binary vector of a protein domains. Broadly, the work done is executed well but conclusions are significantly overstated.*

Response: We agree that limitations of our methodology were not emphasized enough in the previous version.

Action: We have addressed this point in two ways: we have improved the methodology by representing enzymes through UniRep vectors (deep learning representations of the amino acid sequences; l. 181), and we now clearly discuss the limitations of our approach (e.g., l. 332).

2. *As the authors state line ~70 "a machine learning model trained on domain structures will not be able to distinguish [specific molecule binding affinity]; the model will assign the same predictions for both substrates to each of the two proteins". The authors then go and make a model based on domains. While I agree this model will provide a likely generic estimate for a likely biological relevant ligand based on the chemical structure (which is understandable given the dominance of chemical structure in feature importance) it is not going to be predictive of a molecule's affinity to the protein as it will not account for the effects mutations have within a binding site or tertiary effects. It simply will predict the average likely Km level for the class of proteins, which generally can be extracted simply from a simple look-up table where the average Km for a domain annotated (or slightly more elaborated version where MW or LogP or such are incorporated).*

Response: A simple look-up table might indeed work well when experimental estimates exist for the K_M of a domain and a given substrate. However, we aim to provide predictions for cases where it is unknown which domain is responsible for the binding, and where the substrate is not represented at all in the training data. Still, we agree with the Reviewer that the domain representation of enzymes is unsatisfactory, as it does not take differences in amino acid sequences between homologous enzymes into account.

Action: In response to the Reviewer's comment, we have replaced the enzyme representation by protein domains with a deep learning representation of the amino acid sequence, UniRep (Alley et al. 2019). This now allows us to make specific predictions for different sequences belonging to the same enzyme family, and leads to a substantial improvement of the predictions.

3. *Furthermore, the authors seem to have either ignored or been unaware of the decades of research in the field of affinity prediction, the following review (I am not an author or affiliated with any of the authors) nicely summarizes:*
   Comparison Study of Computational Prediction Tools for Drug-Target Binding Affinities, https://www.frontiersin.org/articles/10.3389/fchem.2019.00782/full

   A simple google search "predicting ligand affinity" or "predicting small molecule binding" or related identifies hundreds of manuscripts using ML to predict Ki/Kd/IC50 which in all essence are equivalent to Km being the pseudo-binding constant of an enzyme.

Response: We thank the Reviewer for pointing out this omission.

Action: We now discuss drug target binding affinity (DTBA) predictions in the introduction (l. 45) and compare our prediction quality with that of two DTBA models (l. 308).

4. *Therefore, while this article is interesting and contributes to the field, it should do a better job placing the advance within the field, make more accurate claims about the utility and advance, and is likely more appropriate for a trade journal. I do apologize if this review seems overly harsh, however I was truly excited when I read the title and abstract and found myself deeply frustrated by the time I had concluded reading the article.*

Response: We apologize for having caused this frustration, and we hope that the Reviewer will find the revised manuscript somewhat less disappointing.

Action: See our responses to points 1-3.

Reviewer #3:

In this manuscript, the authors aimed to predict the values of Km from structures of the substrate and the enzyme. They represented the substrate structure with a graph neural network, accounting for comprehensive information of the atoms and their interactions with neighbors. For the enzyme structure, however, they used a much simpler representation: what functional domains exist in the enzyme. Using training data set from the BRENDA database, the authors were able to make predictions of Km values. The results were better than a previous work (Borger et al. 2006).

Given the huge variation of Km values in the BRENDA database - for a typical enzyme in BRENDA, the Km value deviates 3.7-fold from the mean of all the values for that enzyme - the predicted values here on average deviate 4.3-fold and thus seem valuable. Furthermore, with the readily obtainable results from the computational model and the expandability of the model, I think this manuscript is an important contribution to understanding enzymatics at the systems level. I however do have some issues that I like the authors to address.

Response: We thank the reviewer for this positive assessment.

1. *The authors present the model as the main result. However, to most biologists including me, the predicted values of Km are probably most valuable. I thus suggest the authors apply the model to all possible reactions in popular organisms such as human and mouse. Ideally, the results can be made available in the format of website (which can be updated as the model is updated in future). At least an excel file containing the numbers should be included in the supplementary materials.*

Response: We agree that providing predictions for popular model organisms is indeed desirable.

Action: In Dataset S1, we now provide K_M predictions for all reactions in 47 curated genome-scale metabolic models, including those for human, mouse, the yeast *Saccharomyces cerevisiae*, and *E. coli*.

2. *The representation of the enzyme structure seems over-simplified. The author discussed a number of enzyme properties that can be considered when they become more widely available in future. The order of the domains (along the protein sequence) however is available. I wonder if this structurally important information can be incorporated into the model?*

Response: We thank the Reviewer for this suggestion. However, in light of this comment and comments by the other Reviewers, we feel that such a change would still not allow us to adequately represent enzyme amino acid sequences.

Action: In the revised manuscript, we represent enzymes through a deep learning representation of the amino acid sequence, UniRep (Alley et al. 2019). This now allows us to make specific predictions for different sequences belonging to the same enzyme family, and leads to a substantial improvement of the predictions.

3. *Can the authors discuss the applicability of the model to the prediction of Kcat?*

Response: We agree that predicting k_{cat} may be possible with a similar framework; an important difference would be the requirement to represent all substrates (and possibly

products) of the enzyme as simultaneous inputs. While we find this an interesting suggestion, we consider it to be beyond the scope of our analysis and leave it for future work.

Action: We now indicate the applicability of a similar framework to the prediction of k_{cat} in the Discussion (l. 355).

4. *The origins of the Y-axis of the MSE and R2 figures were randomly chosen at the moment. I suggest set all of them to 0.*

Response: While we agree with the reviewer that setting all origins to zero would make it easier to visually compare across figures, the differences between methods in some of the figures are so small that they would be hard to distinguish.

Action: None.

Reviewer #4:

> *The manuscript by Kroll et al. uses a combination of machine and deep learning to predict Km values based on available information on substrate fingerprints. Currently, machine and deep learning are very "hot" topics in all fields. Their contribution to parameter prediction is of particular value to the systems biology community as it offers significant assistance in model parametrization.*
>
> *The framework seems to be well researched and structured and the presentation of the paper is very good. The results also look very promising for Km prediction and it would indeed be very interesting to see results for Kcat parameters in the future. Also the github page with the model information is very practical and very well detailed.*
>
> *A few points should be addressed before acceptance.*

Response: We thank the reviewer for this positive assessment.

Major issues

1. *The test procedure seems a little basic as there is only one test dataset. A full kfold cross validation would be better.*

Response: We agree that performing a *k*-fold cross validation is a more common approach and provides additional information.

Action: We now perform 5-fold cross validations for all analyses (e.g., see Figures 2-4).

2. *It would also be nice to get some empirical investigation as to how robust the results are to different choices of train/validation/test data. It would be interesting to see how much the hyperparameters change, whether the errors are stable etc.*

   Both of these issues could be addressed fairly simply by re-running the analysis on different splits.

Response: We agree that such additional information is valuable.

Action: The 5-fold cross validations we now perform demonstrate that the performance is relatively robust across different splits (Figures 2-4).

3. *The choice about processing the input data (averaging log kms for enzyme/substrate combinations with the same functional domain) isn't well argued for and may have biased the results somewhat. A mentioned in lines 83-86: "Since we are only interested in one KM value for every substrate in combination with a group of enzymes with the same functional domain content (see below), we took the geometric mean if multiple values existed for the same combination of substrate and enzyme domain content."*

   Not sure if this is a good idea - why throw away information like this? The mean is only a good summary statistic if the error distribution is approximately normal (which probably isn't the case here given that BRENDA data tends to be quirky) and even in this case the fact that certain data points represent more observations should be taken into account. Based on the arguments in the paper I think it would have been better to omit this step and allow multiple measurements of the same enzyme/substrate combination.

Response: We agree with the Reviewer that valuable information was lost through the averaging of K_M values across enzymes with the same domain content.

Action: In the revised manuscript, we now retain the original, enzyme-specific data. We further changed the model input to facilitate making enzyme-specific predictions. Enzymes are now represented through a deep learning representation of the amino acid sequence, UniRep (Alley et al. 2019). This change leads to a substantial improvement of the predictions.

4. **** line 375: "We tested the null hypothesis that the median of the absolute errors on the test set..." Why median absolute error not MSE?*

Response: We wanted to use an established statistical test that does not rely on normality assumptions. We found the Wilcoxon signed-rank test appropriate for the task; this test compares median absolute errors.

Action: We clarified the use of the Wilcoxon signed-rank test as the reason for comparing medians in the manuscript (l. 538).

Minor issues

5. *It would be better to package the dataset that was used for the analysis in the paper instead of downloading it from BRENDA for reproducibility purposes. As BRENDA changes periodically, some of the information may be different and it might not be possible to obtain the same results.*

Response: The dataset (together with the scripts used for their assembly) was already provided on github for the previous submission; you can find it here: https://github.com/AlexanderKroll/KM_prediction/tree/master/datasets/BRENDA_data. We apologize for not making that sufficiently clear in the manuscript.

Action: We now explicitly state in the manuscript that the all datasets generated for the manuscript are available together with the Python code (l. 362).

Reviewer #5:

In this manuscript, Kroll et al. describe and train a model to predict Km using machine learning. The authors are correct in presenting and highlighting the problem around the availability and the challenges of calculating km comprehensively, consistency and at scale. I also agree that the correct prediction of Km at scale will enormously benefit other fields so the impact could be huge. I however have significant methodological questions and I am not fully convinced that PLoS Biology is the ideal home for this manuscript. I think the manuscript on its present form is very technical and I am not sure of interest to the broad readership of PLoS Biology. My advice would be to perform major changes to adapt it to a wider audience or to submit to a more technical journal such as PLoS Computational Biology or j chem inf mod. The article is well written and relatively easy to follow. Below I detail my comments and questions:

Response: We thank the reviewer for this positive assessment.

1. ***(a)*** *Regarding the training/test data, the authors use BRENDA which quite a standard database. I however don't see the data being available as supplementary. I understand that there is a script in github to do the preprocessing but since BRENDA can be updated I would make the data preprocessed available as a supplementary for better reproducibility and future comparisons. This would also be of help to the non-computational readers of PLoS Biology.* ***(b)*** *I was a bit surprised to see that the authors don't use also SABIO-RK database, that seems quite a standard in the field. On a quick search they don't seem to include exactly the same information so I wonder why the authors did not use it for model building or testing (see comment below).* ***(c)*** *Finally, I was a bit surprised why the authors don't doo 5- or 10-fold cross validation although I have more questions regarding model testing below.*

Response (a): The dataset (together with the scripts used for their assembly) was already provided on github for the previous submission; you can find it here: https://github.com/AlexanderKroll/KM_prediction/tree/master/datasets/BRENDA_data. We apologize for not making that sufficiently clear in the manuscript.

Action (a): We now explicitly state in the manuscript that the all datasets generated for the manuscript are available together with the Python code (l. 362).

Response (b): Sabio-RK contains much less information than BRENDA, but indeed holds a few hundred K_M values not available from BRENDA. We thank the Reviewer for the suggestion to use this data as an additional test set.

Action (b): We use the non-overlapping data from Sabio-RK as an additional, independent test set, as suggested (l. 220; Figure S1).

Response (c): We agree that a *k*-fold cross validation is indeed standard and provides additional information.

Action (c): We now perform 5-fold cross validation for all analyses (e.g., Figures 2-4).

2. *I think it was a wise decision to use parameters that are broadly applicable to aim for a method that is applicable to any enzyme of any species and a strength as compared to other models that use information that is not broadly available. I liked the idea of using ECFPs and RDKit is indeed a very good standard in chemoinformatics. However, there are many different types of fingerprints so it seems a wasted opportunity to not*

look a little at other fingerprint approaches. I would use at least another fingerprint to compare if you see any differences. In my opinion 2D-pharmacophore fingerprints have shown quite robust results and I think it would be interesting to see if better results are achieved... But if the authors would prefer another fingerprint to compare I would have no objections, I just think it would be nice to see at least another fingerprint tested.

Response: We agree that testing ab additional fingerprint is desirable.

Action: We now added MACCS keys as an additional fingerprint (Figure 2).

3. *Regarding model building, the authors jump straight away to using FCNNs. I think it would be very interesting to see how NNs compare to simpler models. So I would start by implementing a simpler model before jumping to neural networks. Maybe linear modelling (e.g. elastic search) would be a good place to start to be able to assess the added value of using more complex models.*

Response: We agree that testing a simple, linear model is important to provide a baseline against which to compare more complicated models.

Action: In addition to examining the fully connected neural network and the gradient boosting model, we now evaluate the performance of an elastic net (l. 114, Table S3).

4. *The description of the results on graph neural networks was a bit technical. I wonder if it would be too much for a wet-lab reader of PLoS Biology... I would consider simplifying it and/or moving part of it to the results and in the main text focus more on the outcomes of the model and its applicability.*

Response: The description of GNNs was indeed overly technical in the original submission.

Action: We simplified the description as suggested. We restructured the introduction, and we now give only a simple description of the different metabolite fingerprints (including GNNs) in the Results section (l. 133), leaving a detailed description of GNNs to the Methods section (l. 468).

5. *I liked how information was subsequently added on molecular weight, partition coefficient and functional domain to assess the effects in the model.*

Response: We thank the reviewer for this positive comment.

Action: None.

6. *The authors then describe how to predict km for substrates and enzymes not present in the training data. And I get slightly confused and this is highlights the importance of providing the data that was used for model building and testing as a supplementary (see above). The numbers discussed seem really low. Only 3 homologous enzymes with the same substrate were present in the training and test set? And only 9 where the enzyme and substrate are not in the training? then additional trainings were created but again 45 points seems low to me, given there are 5,000 data points. So I think this needs careful design from the outset and more clear explanation. I think the authors should do x-fold cross validation of all the models and these test/training sets should be designed to prevent the same enzymes/homologues and substrates to be*

present in both training and test sets. That would be the fair approach to testing it in my opinion.

Response: As we now use data for individual enzyme-substrate combinations (rather than pooling the data for a given combination of protein domains), these numbers have changed. There are now 664 enzyme-substrate combinations in the test set that do not occur in the training set. For 57 enzyme-substrate combinations in the test set, neither enzyme nor substrate are represented in the training set.

Action: The updated analysis of enzymes and substrates not represented in the training set is found in the section starting l. 228.

7. *I appreciate the authors discuss the MSE differences with previous approaches despite not being possible to do a comprehensive comparison. I wonder why they don't discuss R2 too and there are a couple of previous works that are also not included in this discussion. I think this part should be elaborated more.*

Response: The previous work that we are aware of does not report R^2 values that can be compared to our work.

Action: We now additionally compare the performance of our model to two studies predicting drug target binding affinities (DTBA), which report r_m^2 values (l. 308).

8. *In an ideal world, I would like to see a prospective experimental validation at the end of the paper. I think this is important, particularly if the aim is a general biology journal as PLoS Biology as opposed to a computational journal. I appreciated this may be difficult as measuring Km is not simple and the authors are fully computational. But I wonder if the authors could alleviate this somehow. Maybe using the SABIO-RK data that is not covered by BRENDA as an unseen dataset? Or maybe curation some recent Km literature values that did not make it into BRENDA yet? I think this would be important.*

Response: Experimental measurements of previously undetermined K_M values is indeed beyond our expertise. However, given that the Sabio-RK database contains 274 enzyme-substrate combinations not represented in BRENDA, this appears to provide an even better test case than a few newly measured K_M values.

Action: As suggested, we now compare our predictions to independent, non-overlapping K_M data from Sabio-RK (l. 220).

9. *Finally, I think there are two aspects that would make this far more appealing for a broad biology journal. These would not be necessary for a computational journal but I think they are for PLoS Biology.* ***(a)*** *The first one is data availability. I know the code is in github but experimental scientists that could benefit from your model can't access this easily. I think you should provide the Km predicted with your best model for all enzymes. Of course this not easy to do but maybe you could calculate them for all the enzymes in the most used models and release these data. Alternatively, my preferred option would be that you somehow enabled wet-lab scientist to be able to calculate the km themselves using your method with an easy online application, for example deploying a shiny app.* ***(b)*** *The second aspect is that the paper is very dry on its current form. Could you maybe end with a simple example of use? Maybe an example where the Km is not known for am enzyme and this makes it very challenging to generate a simple model of cellular metabolism. But when you estimate the km using your best model, then you can easily construct a simple metabolism model for this*

enzyme that explain experimental observations. This is just a suggestion but ending the paper with an example of use would make if far less dry and more palatable for non-expert experimental scientists.

Response: We agree that providing K_M estimates for a broad range of enzymes and model organisms is indeed desirable. While providing a shiny app is beyond the scope of our current study, providing predictions for a range of model organisms is indeed possible. We like the idea of supplying a simple use case at the end of the manuscript, which would indeed make it a more enjoyable read especially for many experimental scientists. However, while such use cases undoubtedly exist, we failed to identify an example that is simultaneously simple and illustrative.

Action: As suggested, we now provide K_M predictions for all reactions in 47 curated genome-scale metabolic models, including those for human, mouse, *Saccharomyces cerevisiae*, and *E. coli* (Dataset S1).

10. *In summary, I have enjoyed reading the manuscript and the article is clearly a step forward in Km prediction. I therefore strongly encourage the authors to perform the major revisions suggested or transfer the paper to a computational journal with fewer revisions needed.*

Response: We thank the reviewer for this assessment.

Action: As detailed above, we have made major changes to the manuscript, addressing virtually all concerns raised by the five reviewers.
