# Supplementary material for: Deep learning allows genome-scale prediction of Michaelis constants from structural features
Source: PLoS Biol. 2021 Oct 19;19(10):e3001402. doi: 10.1371/journal.pbio.3001402 (PMC8525774; doi:10.1371/journal.pbio.3001402)
Supplement: Attachment — Submitted filename: Kroll_Response2Rev4.pdf [file pbio.3001402.s012.pdf]

Reviewer #3

The authors have satisfactorily addressed all my concerns.

Action: None.

Reviewer #4:

All points were answered successfully and I have no further comments.

Action: None.

Reviewer #5:

I thank the authors for their hard work addressing nearly all the comments from the 5! reviewers. I think they have done a good job, the paper is now much stronger and I have enjoyed (and learned!) reading the revised version of the manuscript. I therefore recommend this paper for publication. I would finally encourage the authors to apply the model that they have developed, maybe by starting collaborations with metabolite flux scientists that are dry or wet lab. We need good models need to get out there and be used! Congratulations.

Response: We thank the reviewer for this thoughtful and positive assessment.

Action: None.
