# Supplementary material for: Deep learning allows genome-scale prediction of Michaelis constants from structural features
Source: PLoS Biol. 2021 Oct 19;19(10):e3001402. doi: 10.1371/journal.pbio.3001402 (PMC8525774; doi:10.1371/journal.pbio.3001402)
Supplement: Attachment — Submitted filename: Review Plos 2-2021.docx [file pbio.3001402.s010.docx]

The manuscript by Kroll *et al.* uses a combination of machine and deep learning to predict Km values based on available information on substrate fingerprints. Currently, machine and deep learning are very “hot” topics in all fields. Their contribution to parameter prediction is of particular value to the systems biology community as it offers significant assistance in model parametrization.

The framework seems to be well researched and structured and the presentation of the paper is very good. The results also look very promising for Km prediction and it would indeed be very interesting to see results for Kcat parameters in the future. Also the github page with the model information is very practical and very well detailed.

A few points should be addressed before acceptance.

Major issues

1) The test procedure seems a little basic as there is only one test dataset. A full kfold cross validation would be better.
2) It would also be nice to get some empirical investigation as to how robust the results are to different choices of train/validation/test data. It would be interesting to see how much the hyperparameters change, whether the errors are stable etc.

Both of these issues could be addressed fairly simply by re-running the analysis on different splits.

3) The choice about processing the input data (averaging log kms for enzyme/substrate combinations with the same functional domain) isn't well argued for and may have biased the results somewhat. A mentioned in lines 83-86: “*Since we are only interested in one KM value for every substrate in combination with a group of enzymes with the same functional domain content (see below), we took the geometric mean if multiple values existed for the same combination of substrate and enzyme domain content*.”

Not sure if this is a good idea - why throw away information like this? The mean is only a good summary statistic if the error distribution is approximately normal (which probably isn't the case here given that BRENDA data tends to be quirky) and even in this case the fact that certain data points represent more observations should be taken into account. Based on the arguments in the paper I think it would have been better to omit this step and allow multiple measurements of the same enzyme/substrate combination.

4) *** line 375: “*We tested the null hypothesis that the median of the absolute errors on the test set...*”

Why median absolute error not MSE?

Minor issues

It would be better to package the dataset that was used for the analysis in the paper instead of downloading it from BRENDA for reproducibility purposes. As BRENDA changes periodically, some of the information may be different and it might not be possible to obtain the same results.
